# Profit (*p*)-Index: The Degree to Which Authors Profit from Co-Authors

**DOI:** 10.1371/journal.pone.0059814

**Published:** 2013-04-03

**Authors:** Nasir Ahmad Aziz, Maarten Pieter Rozing

**Affiliations:** 1 Department of Neurology, Leiden University Medical Centre, Leiden, The Netherlands; 2 GGZinGeest (Geestgronden), Institute for Mental Health Care, Amstelveen, The Netherlands; Consejo Superior de Investigaciones Cientifics, Spain

## Abstract

Current metrics for estimating a scientist’s academic performance treat the author’s publications as if these were solely attributable to the author. However, this approach ignores the substantive contributions of co-authors, leading to misjudgments about the individual’s own scientific merits and consequently to misallocation of funding resources and academic positions. This problem is becoming the more urgent in the biomedical field where the number of collaborations is growing rapidly, making it increasingly harder to support the best scientists. Therefore, here we introduce a simple harmonic weighing algorithm for correcting citations and citation-based metrics such as the *h-*index for co-authorships. This weighing algorithm can account for both the nvumber of co-authors and the sequence of authors on a paper. We then derive a measure called the ‘profit (*p*)-index’, which estimates the contribution of co-authors to the work of a given author. By using samples of researchers from a renowned Dutch University hospital, Spinoza Prize laureates (the most prestigious Dutch science award), and Nobel Prize laureates in Physiology or Medicine, we show that the contribution of co-authors to the work of a particular author is generally substantial (i.e., about 80%) and that researchers’ relative rankings change materially when adjusted for the contributions of co-authors. Interestingly, although the top University hospital researchers had the highest *h-*indices, this appeared to be due to their significantly higher *p*-indices. Importantly, the ranking completely reversed when using the profit adjusted *h*-indices, with the Nobel laureates having the highest, the Spinoza Prize laureates having an intermediate, and the top University hospital researchers having the lowest profit adjusted *h*-indices, respectively, suggesting that exceptional researchers are characterized by a relatively high degree of scientific independency/originality. The concepts and methods introduced here may thus provide a more fair impression of a scientist’s autonomous academic performance.

## Introduction

In recent years, the numbers and sizes of groups of collaborating scientists have increased dramatically on both national and international level. Although these intensifying scientific collaborations have many obvious and undeniable advantages, at the same time these developments make it harder to discern and quantify each scientist’s individual contribution to the final outcome of a particular project. In the biomedical field the number of authors has increased steadily since 1950, as can be appreciated from the website of the U.S. National Library of Medicine depicting the number of authors per MEDLINE®/PubMed® citation versus year of publication (http://www.nlm.nih.gov/bsd/authors1.html). Even papers with over hundred authors are increasingly more common, especially in the fields of genomics and proteomics [1]. Therefore, in an era where scientists increasingly need to compete with each other to obtain funding, there is a need for more equitable and fair measures of comparison [1], [2].

The number of publications and the citation frequency are generally regarded indicative of the scientific merits of an individual author and are employed by various citation metrics, the most widely applied of which is the *h*-index: a scientist with an index of *h* has published *h* papers each of which has been cited at least *h* times [3], [4]. However, currently the quantification of citations occurs *irrespective* of both the number of authors on each manuscript and the ranking of each author on the author’s list [3], [4]. While, at least in the biomedical field, it is generally appreciated that the first and last authors have contributed the most to a particular work, and conversely, the authors who are somewhere in the middle have contributed the least, this information is not accounted for in the current counting algorithms of citations [5]. This shortcoming is all the more relevant given the recent expansion in the number of co-authors on individual publications, who have not necessarily contributed substantively to the publication [1]. Modifications of the *h*-index to address the effects of co-authorships have been proposed before [6]–[11]. However, these modifications allocate credit equally among authors irrespective of the author sequence [7], [9], disproportionally privilege the first or the corresponding author [8], [11], or make assumptions about the underlying citation distributions and require iterative calculations and information on the citation tracks of all the co-authors [10]. Moreover, these previous modifications employ arithmetic, geometric or fractional counting of citations, which are less robust than harmonic weighing algorithms for bibliometric analysis [12], [13]. In addition, to the best of our knowledge, there have been no previous reports on metrics which are specifically designed to measure the contribution of others/co-authors to the academic performance of a given author.

Given the aforementioned considerations, in this paper we will derive a simple harmonic weighing algorithm which can be used to weigh each publication simultaneously for both the number of authors and the rank of each author on the author’s list. We demonstrate that accounting for this additional piece of information contained within each paper can have dramatic consequences for the scientific ranking of a given author and will allow for derivation of metrics for estimating a researcher’s individual merits by accounting for the contribution of others to his or her work.

## Methods

### Derivation of a Relative Weighing Factor

For weighing a given publication simultaneously for both the number of authors and the rank of the author among the paper’s authors, we propose a weighing factor (*w*) consisting of at least two parts: a fixed part *A* and a variable part *B*, the sum of which should be smaller than or equal to 1, the maximum weighing factor. That is:
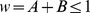



We will let the fixed part *A* represent the proportion of the weighing factor that is determined by the total number of authors on the paper. The most straightforward way to weigh for the total number of authors (*n*) on a paper is to define
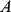
simply proportional to the inverse of 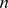, i.e.:
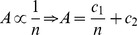
(1)with 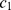 and 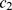 being some constants.

We will let the variable part *B* represent the contribution of the rank of the author to the weighing factor. In order to weigh for the ranking of the author (*i*), we will assume that by definition the first and the last author should get the maximum weight (i.e. 1), and that the weights should proportionally decrease to zero for author ranks at the median. Therefore, the most simple way to define *B* is to let it be proportional to the relative distance from the median, i.e.:
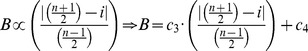
(2)with 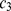 and 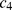 being some constants.

For the sake of simplicity we will assume that 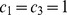, and 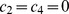. Because 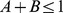, by combining equations (1) and (2) we will get:
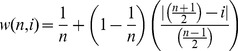
(3)Which can be simplified to:



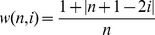
(4)Thus, the formula is so constructed that 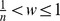, whereby in the case of the first and the last author *w* always will equal 1, whereas in the case of authors near the median *w* will approach 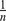.

### Derivation of an Absolute Weighing Factor

Although formula (4) will yield an estimate of the relative contribution of a specific author for a particular paper, for comparisons between papers with different numbers of co-authors a measure of ‘absolute’ contribution is needed. For example, intuitively, a monograph by a particular author should weigh more than a first-author paper by the same individual but with multiple co-authors. To accomplish this feat, *w* should be normalized for the total conjoint effort that has gone into a given paper. This can readily be achieved by dividing *w* by the total sum of the ‘relative’ weights of all the co-authors of a given paper, i.e.:
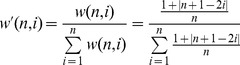
(5)


Since:
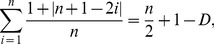
(6)with 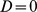 if *n* is an even number, and 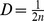 if *n* is an odd number (note that *D* will approach zero for large *n*), equation (5) can be simplified to:



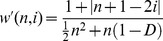
(7)Thus, 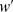 can be regarded as an estimate of the fraction of a given paper which is solely attributable to the efforts of a single author. That is, 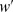 will equal 1 if and only if the paper is a monograph and will proportionally decrease as the number of co-authors increases or as the rank of the author approaches the median.

### Monograph Equivalents and the Profit-index

By calculating 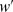 for each paper on an author’s publication list and summing over all these 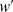
s, one can thus yield an estimate of the equivalent of the author’s publications in ‘monographs’, which we will call the “number of monograph equivalents (*M*)”, i.e.:
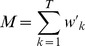
(8)


Here *T* equals the total number of papers on an author’s publication list. Now we will define the ‘profit (*p*)-index’ as the relative contribution of other individuals to the total publication record of a given author, i.e.:
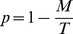
(9)


The *p*-index will thus vary between 0 and 1 (i.e. 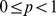), with higher values indicative of a greater contribution of other individuals to an author’s track record. Thus, in this way the *p*-index could be used as an approximate estimate of the degree to which authors profit from the contributions of their co-authors.

### Adjusting Traditional Metrics: the Adjusted h-index and the Profit h-index

Based on the metrics described above, other citation derived measures such as the *h*-index could also be further adjusted and expanded. This can directly be accomplished by weighing the number of citations each paper has received by 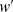
and then calculating the *h*-index using these ‘adjusted citation numbers’. This adjusted index, the 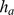-index, will thus estimate the *h*-index of a given author in the case he/she would have only produced monographs. Along similar lines of reasoning one could also construct a ‘profit *h*-index (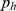)’ as follows:
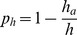
(10)


Thus 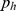 could be used as a rough estimate of the relative contribution of other individuals to the ‘traditional’ *h*-index of a given author.

### Illustrations

In order to characterize the above derived metrics further, and compare and relate them to more traditional metrics, we calculated all these metrics for all university professors in the biomedical field employed at our institute, the Leiden University Medical Centre in Leiden, the Netherlands. The Leiden University is the oldest university in the Netherlands and according to the 2012 Academic Ranking of World Universities (ARWU) it is ranked 73^th^ among all world universities, and is the highest ranked Dutch university in the field of Clinical Medicine and Pharmacy (http://www.arwu.org). According to the *Times Higher Education* World University Rankings, it is the highest ranked Dutch university in 2012–13 (http://www.timeshighereducation.co.uk/world-university-rankings/2012-13/world-ranking). We retrieved a list containing names of all these individuals from the university’s website (http://www.lumc.nl/0000/12296/hoo/) on April 12^th^, 2012 (n = 163). Two of these subjects were excluded from further analyses as they predominantly appeared to have published in Dutch journals, in the fields of medical ethics and anthropology, not included in the Science Citation Index.

In order to assess whether there were any differences between the bibliographical metrics of the top 15 researchers from our institute (i.e. those with the highest *h-*indices) and other excellent biomedical researchers we employed the following strategy: We defined excellent national researchers as those who have been awarded the Spinoza prize, which is regarded as the highest scientific distinction in the Netherlands and is colloquially referred to as the ‘Dutch Nobel prize’, in the field of Life Sciences. A list of the Spinoza laureates was retrieved from the website of the Netherlands Organization of Scientific Research (http://www.nwo.nl/nwohome.nsf/pages/NWOP_8G4B8S) on May 6^th^, 2012. In order to increase comparability we only included those individuals who had won the prize in 2001 or later (n = 12). One individual among the Leiden University’s top 15 was also a Spinoza laureate, therefore, this individual was excluded from the analysis when comparing the Leiden with the Spinoza group. We defined excellent biomedical international researchers as those who have been awarded the Nobel prize in Physiology or Medicine. We identified these individuals from the official website of the Nobel prize committee (http://www.nobelprize.org/nobel_prizes/medicine/laureates/; accessed on April 30^th^, 2012), again including only those who have been awarded the prize in 2001 or later (n = 27). We specifically postulated that the highest national and international scientific recognition could be used as an indicator of originality.

During the period between April 12^th^ until May 10^th^ 2012, we retrieved the publication and citation records from the *ISI* Web of Knowledge (http://apps.webofknowledge.com), and calculated all the metrics described hitherto, summaries of which are presented in the results section. Results are expressed as mean ± standard error (SE) or, in case of a non-normal distribution or small sample size, as median (25^th^–75^th^ percentiles). Because of relatively small group sizes the omnibus non-parametric Kruskal-Wallis analysis of variance by ranks was used to assess potential intergroup differences in medians, while pairwise Mann-Whitney *U* tests were applied to identify those medians which caused the difference. Spearman’s ρ was used to assess all correlations. All tests were two-tailed and significance level was set at p<0.05. Statistical analyses were performed using PASW Statistics (release 18.0.0., SPSS Inc., Chicago, IL).

## Results

### The Profit Index is Generally High

**Figure 1:**
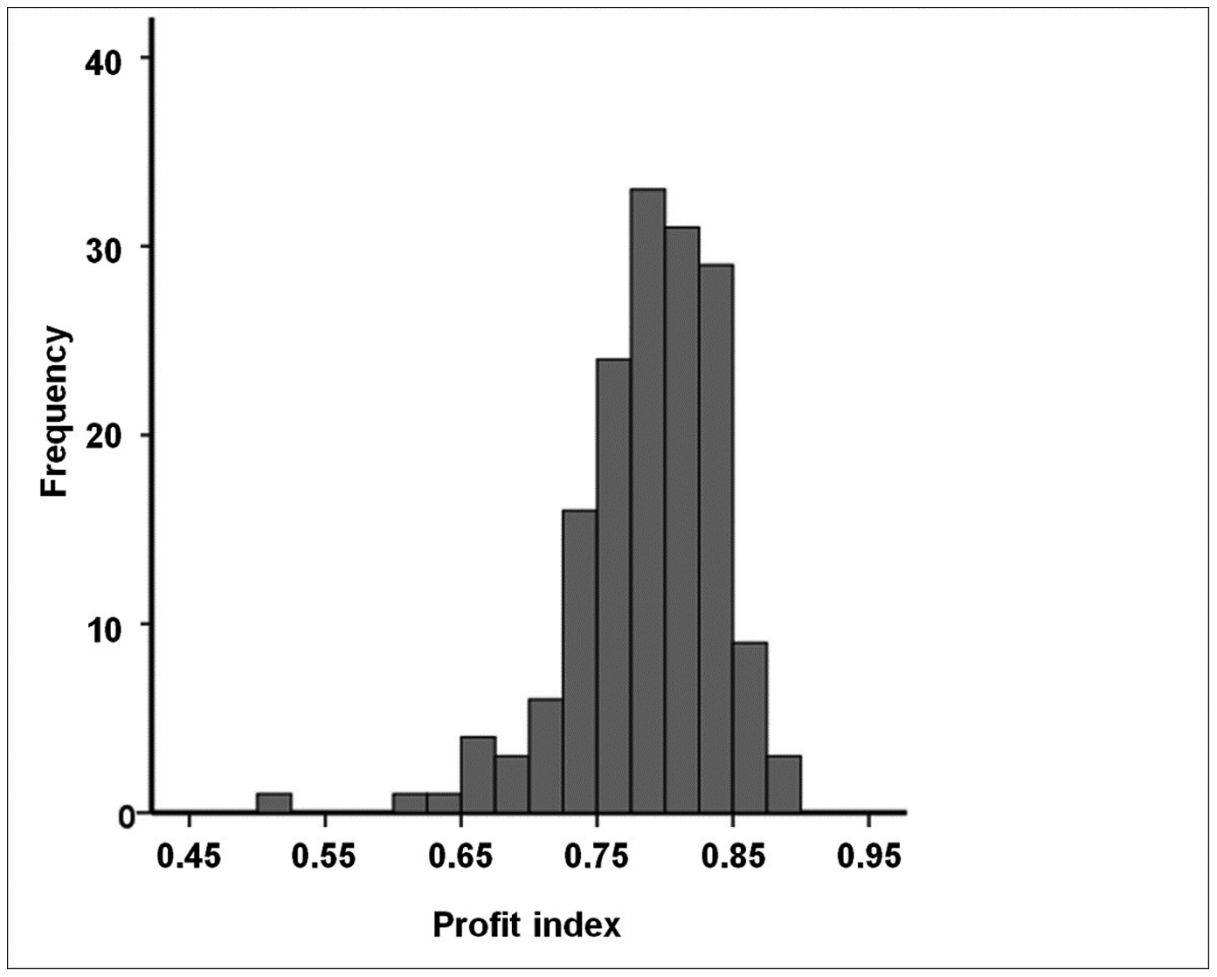
The distribution of the profit (*p*)-index. Among the University Medical Centre researchers (n = 161), the *p*-index was approximately normally distributed with a mean of 0.79 and a standard deviation of 0.05 (see also **Table 1**).

A total of 161 subjects were included whose characteristics are displayed in **Table 1**. The mean number of publications per subject was 232.50±17.89. However, the number of ‘monograph equivalents’ was substantially lower (46.91±3.47), resulting in a relatively high mean *p*-index (0.79±4.2 × 10^−3^) with a relatively narrow range of variation (**Figure 1** **and** **Table** **1**). This suggests that the contribution of others to the track record of a particular author is generally enormous (i.e. about 80%). Similarly, the mean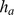-index was considerably lower (13.02±0.57, range 2 to 42) than the mean *h*-index (34.60±1.44, range 3 to 83), likewise resulting in a comparatively high 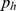-index (0.61±6.6 × 10^−3^). The 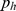-index was about 25% lower than the *p-*index, suggesting that the contributions of others to the most ‘influential’ papers of a given author are less than to his total track record, albeit still very substantial.

**Table 1:** Characteristics of 161 University Medical Centre researchers.

	Mean	*SE*	Median	Mode	*SD*	Minimum	Maximum
No. of papers per subject	232.5	17.88	153	117^a^	226.84	9	1556
Total no. of citations per subject	5940.57	511.32	3966	751^a^	6487.86	28	34232
No. of co-authors per paper	7.12	0.19	6.75	3.08^a^	2.39	3.08	22.97
*h-*index	34.60	1.44	32	28^a^	18.26	3	83
*h_a_*-index	13.02	0.57	12	10^a^	7.22	2	42
*p*-index	0.79	0.0042	0.794	.52^a^	0.05	0.52	0.90
*p_h_*-index	0.61	0.0066	0.63	.60^a^	0.08	0.29	0.79
No. monograph equivalents	46.91	3.47	31.55	2.55^a^	43.99	2.55	254.38

a Multiple modes exist. The smallest value is shown. *SE:* standard error of the mean; *SD*: standard deviation; *h_a_* –index: profit adjusted *h*-index; *p*-index: profit index; *p_h_*-index: profit *h*-index.

### The Profit Index in Relation to the Number of Co-authors, the Number of Publications and the h-index

**Figure 2:**
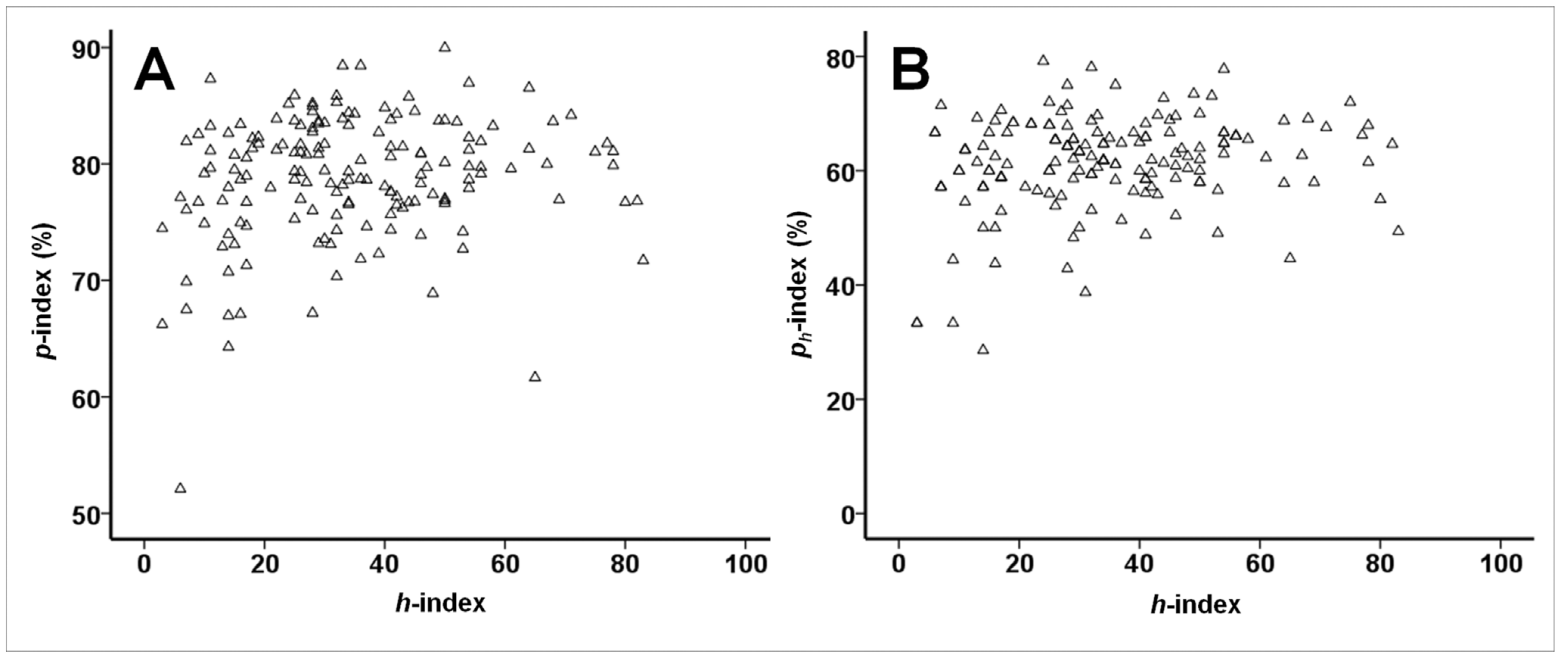
The relative contribution of co-authors. Among the University Medical Centre researchers (n = 161), there was a non-significant trend for the association between the *p*-index and the *h*-index (ρ = +0.15, p = 0.067), while the *p_h_-*index and the *h*-index were only weakly associated (ρ = +0.17, p = 0.031), suggesting that the relative contribution of co-authors to the work of an author is relatively stable over his/her scientific career.

As expected, the number of co-authors was strongly associated with both the *p*-index (ρ = +0.69, p<0.001) and the 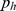-index (ρ = +0.52, p<0.001). The association between the number of publications and the *p*-index (ρ = +0.20, p = 0.010) or the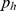-index (ρ = +0.27, p = 0.001) was considerably weaker. There was a non-significant trend for an association between the *p*-index and the *h*-index (ρ = +0.15, p = 0.067), while the 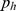-index and the *h*-index were only weakly associated (ρ = +0.17, p = 0.031). By assuming that the number of publications or the *h*-index could serve as rough proxies of the length of the scientific career, these findings thus suggest that the relative contribution of co-authors to the work of an author is relatively stable over his/her scientific career (**Figure 2**). Furthermore, there was a strong association between the *p*-index and the 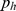-index (ρ = +0.69, p<0.001), and as expected, neither the *p*-index nor the 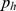-index were associated with the number of monograph equivalents or the 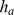-index (all p>0.14).

### Scientists’ Relative Rankings change Substantially when using the Profit Adjusted h-index

Two scenarios are displayed in **Table 2**: In the second column the 161 scientists are ranked according to their *h*-index, whereas in the third column they are ranked according to their profit adjusted *h* (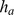)-index (only the top 15 subjects are shown). It is immediately clear from this table that the relative rankings of these scientists are greatly dependent on the indicator which is used, i.e. the *h-* or the 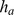-index. When using the 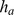-index only 6 scientists retained their original relative ranking based on the *h-*index, whereas all others changed position. The mean absolute change in ranks was 11.0±0.91 (or 11/161 ≈ 7%), with a standard deviation of 11.5. Notably, there was an individual who descended 61 positions on the rankings list. These figures indicate that on an individual basis the ranking can greatly be affected by the extent to which authors rely on the work of others.

**Table 2:** Scientists’ relative rankings change substantially when using the profit adjusted *h*-index.

Ranking among 161 peers	Based on the *h*-index		Based on the *h_a_-*index	
**1**	*Subject 1*	**83**	*Subject 1*	**42**
**2**	*Subject 2*	**82**	*Subject 3*	**36**
**3**	*Subject 3*	**80**	*Subject 12*	**36**
**4**	*Subject 4*	**78**	*Subject 5*	**30**
**5**	*Subject 5*	**78**	*Subject 2*	**29**
**6**	*Subject 6*	**77**	*Subject 9*	**29**
**7**	*Subject 7*	**75**	*Subject 13*	**27**
**8**	*Subject 8*	**71**	*Subject 26*	**27**
**9**	*Subject 9*	**69**	*Subject 6*	**26**
**10**	*Subject 10*	**68**	*Subject 4*	**25**
**11**	*Subject 11*	**67**	*Subject 11*	**25**
**12**	*Subject 12*	**65**	*Subject 8*	**23**
**13**	*Subject 13*	**64**	*Subject 15*	**23**
**14**	*Subject 14*	**64**	*Subject 27*	**23**
**15**	*Subject 15*	**61**	*Subject 41*	**22**

Two scenarios are displayed here: In the second column the 161 University Medical Centre scientists are ranked according to their *h*-index, whereas in the third column they are ranked according to their profit adjusted *h* (*h_a_*)-index (only the top 15 subjects are shown). Note the substantial changes in the rankings when the *h_a_*-index is used instead of the *h*-index.

*h_a_*-index = profit adjusted *h*-index.

### The Profit (p) Index is Relatively Low in Spinoza and Lowest in Nobel Laureates: a Measure of Originality?

**Figure 3:**
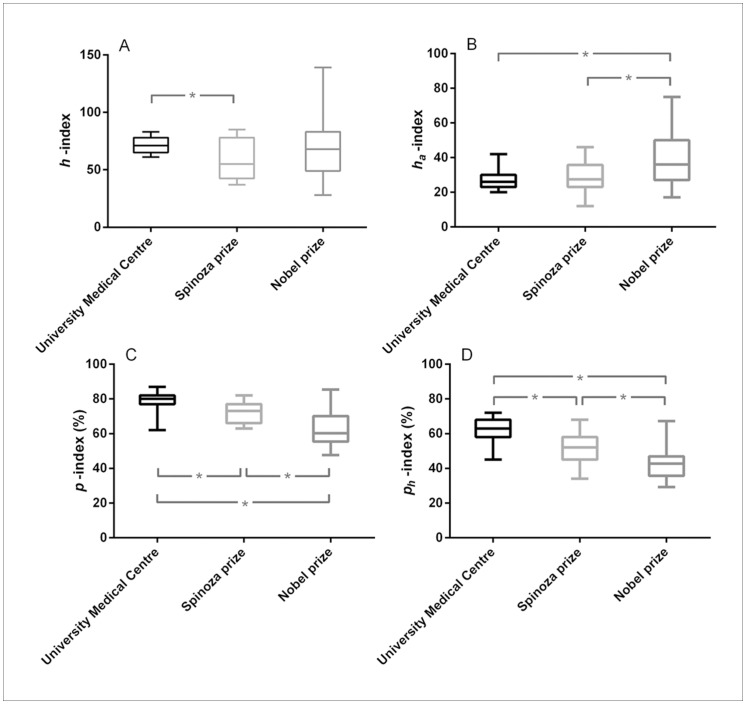
Comparison between University Medical Centre researchers (top 15 with highest *h* -index), Spinoza laureates and Nobel laureates in the biomedical field with respect to different citation metrics. The horizontal lines within the boxes represent the median values, the edges of the boxes indicate the interquartile range, and the outer horizontal lines indicate the minimum and maximum values. Data are given for: **A.** *h*-index; **B.** profit adjusted *h*-index (*h_a_*-index); **C.** profit index (*p*-index); **D.** profit *h-*index (*p_h_-*index). * Denotes p-values smaller than 0.05. P-values were calculated using the Mann-Whitney *U* test and indicate differences in median values. Please refer to Table 3 for the statistical outcomes of the other comparisons.

Results of the comparisons between Leiden researchers (top 15 with the highest *h-*index) and Spinoza and Nobel laureates are presented in **Table 3**. As can be judged from this table the top Leiden researchers had actually a higher *h*-index than both the Spinoza and Nobel laureates, although only the difference with the Spinoza laureates reached statistical significance (p = 0.025). However, the reverse held true for the 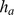-index which was lowest among the Leiden researchers and highest among the Nobel laureates (p = 0.008 for Leiden vs. Nobel laureates). These differences became even more marked when evaluating the profit indices (**Table 3**). Both the *p*- and the 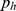-index were highest among the Leiden researchers, somewhat lower among the Spinoza laureates and lowest among the Nobel laureates (p≤0.012 for all pairwise comparisons, **Figure 3**). It is also interesting to note that the Nobel laureates had the least number of co-authors on their manuscripts. As the 15 top Leiden researchers were actually selected based on their high *h*-index and given the significant association between the *h*-index and *p*-index (as described above), we wondered whether these results would change if we would select another group of researchers at the median from the Leiden group (i.e. ranks 74 to 88 according to their *h*-index). However, doing so did not change any of the significances except the *h-*index which was now lowest in the 15 Leiden researchers near the median (data not shown).

**Table 3:** Comparisons between University Medical Centre researchers (top 15 with highest *h*- index) and Spinoza and Nobel laureates in the biomedical field.

	University Medical Centre researchers (n = 15)	Spinoza laureates (n = 12)	Nobel laureates (n = 27)
Total no. of citations	22679^a^ (18897–25554)	11528a, c (7247–24269)	21078^c^ (14531–34199)
No. co-authors	7.1a, b (6.3–8.2)	6.3a, c (5.6–6.8)	4.5b, c (4.0–5.8)
*h*-index	71^a^ (65–78)	55^a^ (42.5–78)	68 (49–83)
*h_a_*-index	26^b^ (23–30)	27.5^c^ (23–35.8)	36b, c (27–50)
*p*-index	0.80a, b (0.77–0.82)	0.73a, c (0.66–0.77)	0.60b, c (0.55–0.70)
*p_h_*-index	0.63a, b (0.58–0.68)	0.52a, c (0.45–0.58)	0.43b, c (0.36–0.47)
Monograph equivalents	134a, b (85–180)	73^a^ (60–104)	99^b^ (48–151)

Values are indicated as median (25^th^–75^th^ percentiles). The Mann-Whitney *U* test was used to assess differences in medians:

a p<0.05 for the comparison between University Medical Centre researchers and Spinoza laureates,

b p<0.05 for the comparison between University Medical Centre researchers and Nobel laureates,

c p<0.05 for the comparison between Spinoza and Nobel laureates. *h_a_* –index: profit adjusted *h*-index; *p*-index: profit index; *p_h_* –index: profit *h* –index.

## Discussion

In scientometrics and bibliometrics research has traditionally focused on developing metrics for estimating an author’s academic performance by analyzing his or her publications, and citations of thereof, as if these were solely attributable to the author. However, this approach completely ignores the often substantive contributions of others to the academic performance of a given author which can lead to misjudgments about the individual’s own scientific merits, and consequently to misallocation of funding resources and academic positions. This problem is becoming the more urgent in the biomedical field where the number of collaborations is growing rapidly, making it increasingly harder to identify and support the best scientists among their peers. Unfortunately, the converse is also true: it is becoming increasingly difficult to identify co-authors who benefit from the original ideas and efforts of others without contributing substantially themselves. Despite these practices being all but apparent to the intimate insiders, currently there is no measure to quantify to which degree authors actually profit from the work of their co-authors.

In order to at least partially overcome the aforementioned problems, in the present article we first introduced a weighing factor for weighing a publication for both the number of authors and the sequence of authors in which they appear on the publication. We demonstrated that widely used metrics such as the *h*-index can be adjusted by using this weighing factor and thereby become more accurate estimates of the academic performance of a given author. Second, from the weighing factor we derived a measure estimating the degree to which authors profit from co-authors, called the ‘profit (*p*)-index’, simply defined as the relative contribution of co-authors to the total work of an author. By using publication and citation data from samples of researchers from a large Dutch University hospital, Spinoza Prize laureates (the most prestigious Dutch science award), and Nobel Prize laureates, we showed that the contribution of co-authors to the track record of a particular author is generally substantial and that researchers’ relative rankings change materially when using these adjusted metrics (in this case the profit adjusted *h*-index as opposed to the classic *h*-index). Notably, it was striking to discover that although the top University hospital researchers had actually higher *h-*indices than both Spinoza and Nobel laureates, this difference appeared to be mainly due to a comparatively high degree of reliance on co-authorships as evidenced by their significantly higher *p*- and 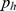-indices. Importantly, the differences completely reversed when using the profit adjusted *h*-indices, with the Nobel laureates having the highest, the Spinoza Prize laureates having an intermediate, and the top University hospital researchers having the lowest profit adjusted *h*-indices, respectively. This finding is crucial as it strongly suggests that exceptionally esteemed researchers are characterized by a relatively high degree of scientific independency/originality (i.e. relatively low *p*- and 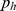-indices), thereby providing a partial answer to the more-than-one-century-old question of what sets the Nobel laureates apart from other excellent scientists. It should be noted though that the Nobel laureates analyzed in this study are probably older and hence likely to have worked in an era with smaller groups of collaborators, which might also have contributed to their lower *p*-indices. However, this latter hypothesis cannot account for the differences between the University hospital researchers and the Spinoza Prize laureates since the Spinoza Prize is only awarded to active researchers and we only selected laureates from 2001 onwards.

As indicated in the introduction section, modifications of the *h*-index and fractional counting of publications or citations to address the effects of co-authorships have been proposed before [6]–[11]. However, these modifications allocate credit equally among authors irrespective of the author sequence [7], [9], disproportionally privilege the first author [8], [11], or make assumptions about the underlying citation distributions and require iterative calculations and information on the citation tracks of all the co-authors [10]. Perhaps the approach proposed by Zhang [11] most closely resembles our algorithm. Nevertheless, there are several important differences between our algorithm and this approach. First, Zhang’s approach assumes weight coefficients for the first and the corresponding author of 1, and assumes that the contribution of the *k-*th author is proportional to 1/*k*. This approach is problematic because frequently the first author is also the corresponding author, and by convention, the most senior author who usually has conceived and supervised the experiments is often the last author on the author’s list (at least in biomedical research). However, according to Zhang’s approach the last author would only get 1/*k* of the credit, which is an unrealistic assumption. In addition, this approach is also problematic in a practical sense because the corresponding author is not indicated in the *ISI* Web of Knowledge, making it very cumbersome to empirically evaluate Zhang’s algorithm. Finally, and perhaps most importantly, the concept of the *p*-index, regardless of the precise weighing algorithm used to derive it, as a measure of what others have done for a researcher, has not been reported before.

There has been some concern about potential biasing effects of preferential self- and co-author citations in bibliometric analyses [14]. However, a more recent study using a large body of citation data from the Thomson Scientific’s Web of Science for the period 1945–2008 failed to provide conclusive support for the existence of such ‘citation cartels’ [15]. Nevertheless, let us assume that for whatever reason (e.g. due to preferential self- and co-author citations) an author would have received more citations than he or she would have deserved in fairness. Even then this would not have influenced the *p*-index as it only depends on the number of co-authors, the rank of the author on each paper and the total number of publications, regardless of the exact number of citations. On the other hand the 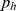-index could in theory be influenced by the number of citations, becoming lower with a higher number of citations if and only if increased citations result in a higher *h_a_* –index but an unchanged *h*-index. In all other cases increased citations will result in proportional increases of the *h_a_* –index and the *h*-index, leaving the 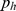-index unaltered. As the University hospital researchers had the most co-authors (Table 3), potential preferential self- or co-author citations would be expected to be most pronounced in this group. Assuming that such an effect would indeed have influenced their 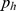-indices, this would mean that in reality the average 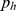-index of this group would have been even higher, and consequently, the difference with the Spinoza and the Nobel laureates even greater.

A crucial assumption in the definition of our citation weighing algorithm is that the sequence in which authors are listed is associated with their respective contribution to the publication. Admittedly, no generally approved assessment of author contribution exists. However, when authors in biomedical research are scored for various forms of participation, the first and last author indeed generally are the principal contributors to the article, while intermediate authors have contributed less [5]. In order to remedy this issue some have proposed to request the authors to rank the authors’ list according to each author’s contribution [16]. We propose to go even one step further and simply request the authors to provide a numerical representation of the contribution of each author to the total body of work behind a publication, just making explicit a widely employed, though often veiled, process which underlies the sequence in which the authors appear on a current paper. Although the modern practice in biomedical journals to chronicle each author’s contribution is a good qualitative representation thereof, a numerical representation will render (semi-)quantitative weighing for co-authorships both much more accurate and easier to standardize by means of automatically computed algorithms. For the present, the applicability of our weighing algorithm (sensu stricto) is thus limited to biomedical sciences and disciplines with comparable conventions of author placement and do not apply to research disciplines with alternative conventions of authorship placement, as for example in mathematics in which authors are often listed in alphabetical order (“http://www.ams.org/profession/leaders/culture/CultureStatement04.pdf”). However, it is relatively easy to adapt our weighing algorithm so as to incorporate disciplines with alternative conventions of authorship placement provided that information about co-authors’ contributions can be extracted from author ranks or in instances where this information is explicitly mentioned. Moreover, regardless of the precise weighing algorithm the concept of the profit index sensu lato, i.e. as an estimate of what others have done for an author, can readily be generalized to other disciplines as long as the co-authors’ contributions could somehow be quantified. It should be further noted that although we have used our citation weighing algorithm to adjust the *h*-index for co-authorships, in principle any bibliographical metric, such as the *g*-index [17] and the *e*-index [18], could be adjusted in a comparable manner. Similarly, it would also be interesting to assess whether co-author-adjusted citation metrics would be better predictors of future scientific success than the unadjusted ones [19].

### Conclusions

In conclusion, in the present article we have introduced a simple harmonic weighing algorithm for correcting citations and citation-based metrics such as the *h-*index for co-authorships. This weighing algorithm can account for both the number of co-authors and the sequence of authors on a paper. We then derived a measure estimating the degree to which authors profit from co-authors, called the ‘profit (*p*)-index’, which simply equals to the contribution of co-authors to the scientific work of a given author. By using citation data from various researchers we found that the contribution of co-authors to the track record of a particular author is substantial and that researchers’ relative rankings change materially when adjusted for the contributions of their co-authors. It was especially noteworthy that the Nobel laureates in physiology and medicine had significantly lower *p*-indices compared to other excellent biomedical researchers, suggesting a relatively high level of independency/originality. We feel that these co-authorship-adjusted metrics may thus provide a more fair impression of the autonomous academic performance of a particular scientist.

### Relative Contributions

NAA/MPR: 2/1.
